# Center of Pressure Measurement Sensing System for Dynamic Biomechanical Signal Acquisition and Its Self-Calibration

**DOI:** 10.3390/s26030910

**Published:** 2026-01-30

**Authors:** Ni Li, Jianrui Zhang, Keer Zhang

**Affiliations:** College of Intelligent Manufacturing, Long Dong University, Qingyang 745000, China

**Keywords:** bipedal robots, center of pressure, measurement sensing system, online self-calibration, ground reaction force

## Abstract

The development of highly dynamic bipedal robots demands sensing capable of capturing key contact-related signals in real time, particularly the Center of Pressure (CoP). CoP is fundamental for locomotion control and state estimation and is also of interest in biomedical applications such as gait analysis and lower-limb assistive devices. To enable reliable CoP acquisition under dynamic walking, this paper presents a foot-mounted measurement system and an online self-calibration method that adapts sensor scale and bias parameters during locomotion using both external foot sensors and the robot’s proprioceptive measurements. We demonstrate an online self-calibration pipeline that updates foot-sensor scale and bias parameters during a walking experiment on a NAO-V5 platform using a sliding window optimization. The reported results indicate improved within-trial consistency relative to an offline-calibrated reference baseline under the tested walking conditions. In addition, the framework reconstructs a digitized estimate of the vertical ground reaction force (vGRF) from load-cell readings; due to ADC quantization and the discrete offline calibration dataset, the vGRF signal may exhibit stepwise behavior and should be interpreted as a reconstructed (digitized) quantity rather than laboratory-grade continuous force metrology. Overall, the proposed sensing-and-calibration pipeline offers a practical solution for dynamic CoP acquisition with low-cost hardware.

## 1. Introduction

Bipedal robots are expected to perform various tasks through dynamic walking. Achieving stable dynamic locomotion is a prerequisite for accomplishing these tasks, and many humanoid control frameworks rely on CoP-based stability criteria as a practical guarantee of balance. Accurate and real-time CoP sensing is therefore essential for gait planning, whole-body balance control, and state estimation in humanoid robots [1,2]. This criterion, rooted in biomechanics, relies on the precise acquisition and processing of the CoP signal—a dynamic physical parameter whose accurate measurement is equally critical in biomedical fields such as clinical gait analysis and the control of lower-limb assistive devices. In gait planning, CoP is constrained to be within the stabilizing convex envelope to ensure the stability of the motion trajectory [3,4,5]. In whole-body motion control, CoP is fed back to the dynamic balance controller to give the robot the ability to push for recovery [6] and adaptive ground [7]. In state estimation, CoP is applied to the center of mass [8,9,10] and external force [11,12,13] state estimation to enable the controller to obtain an accurate estimate of its own motion state. Therefore, the reliable measurement and real-time processing of the CoP signal are of paramount importance for bipedal motion, mirroring its significance as a core biomechanical variable in human movement studies.

However, on small- and medium-sized platforms, CoP sensing hardware is often constrained by cost, weight, and long-term drift, making reliable calibration under dynamic walking a key practical challenge addressed in this work. For CoP measurement, there are two main methods, namely the method using force/torque sensors (F/T sensors) and the method using Force Sensing Resistors (FSRs) [14,15,16]. Among them, F/T sensors have higher measurement accuracy and can measure the CoP position more accurately and thus have been widely used in various large bipedal robot platforms, such as the Wabian series of bipedal robots developed by Waseda University in Japan [17], the HRP series developed by the National Institute of Advanced Industrial Science and Technology (AIST) in Japan [18], the bipedal robots Johnnie [19] and LOLA [20] developed by the Technical University of Munich in Germany, the bipedal robots DLR-Biped [21] and TORO [22] developed by the German Aerospace Center (DLR), as well as the iCub bipedal robot developed by the Italian Institute of Technology (IIT) [23]. Notably, the latest model in the HRP series, HRP-5P, is equipped with F/T sensors on its hands to measure six-axis force/torque data during hand-environment interactions [24]. However, F/T sensors have the disadvantages of higher weight and higher cost. With the trend of bipedal robots toward lightweight design and low-cost production, F/T sensors are difficult to be applied on small- and medium-sized bipedal robots [9,11,12]. Therefore, FSRs have been more widely used on small- and medium-sized robots (e.g., Nao robot [25]). However, compared to F/T sensors, FSRs have lower measurement accuracy and repeatability, suffer from resistive drift and hysteresis, and need to be well calibrated to achieve accurate measurement of CoP. This calibration challenge is a common issue in biomedical signal acquisition systems, where sensor performance must be maintained over time and under varying conditions. In the design of CoP measurement sensing system, Shayan et al. [26] proposed a pressure measurement device based on pneumatic sensors for the Nao robot. Kwon et al. [27] designed a foot pressure measurement system using custom polymer materials. Almeida et al. [28] and Suwanratchatamanee et al. [29] developed foot pressure sensing devices incorporating two-layer and three-layer sensor configurations, respectively.

In terms of the self-calibration of the CoP measurement sensing system, Han et al. [30] proposed an offline manual calibration method for CoP based on regular least squares, which minimizes the error between the measured and true values. However, manual calibration requires human intervention and significant time cost. In addition, the results obtained from offline manual calibration cannot be adaptive according to the actual state of the robot and the environment it is in. For FSRs, sensor measurements tend to drift with subtle changes in mechanical structure or temperature during robot motion. This leads to manual calibration results that become progressively unavailable as the robot moves. Therefore, there is a need to design a self-calibration method for CoP measurements so that the sensing measurement system adapts itself to environmental changes during motion. To address this problem, Han et al. [31] designed a self-calibration method for bipedal static motion CoP measurement using the offline manual calibration method in [30] as a benchmark. The method assumed that the robot motion was in a quasi-static state, i.e., the projection of the center of mass on the ground coincided with the CoP in real time, and self-calibrated the CoP measurement using the real-time center of mass position measurements. This method is the first to self-calibrate the CoP measurements, but it is limited to bipedal static motion and only considers the case of a bi-supported robot. In biped dynamic motion, the projection of the center of mass on the ground does not coincide with the CoP in real time due to the center of mass acceleration, and the CoP only needs to be located within the contact convex envelope of the ground to ensure the stable walking of the robot. In the actual working scenarios of bipedal robots, most of the motions are dynamic, and only a few standing postures satisfy the static motion assumption. Therefore, further consideration needs to be given to the self-calibration of the CoP sensing and measuring system in dynamic bipedal motion scenarios.

In this paper, a dynamic walking CoP measurement sensing system for a biped robot and its self-calibration method are proposed. This work targets practical drift and inter-channel mismatch of foot-mounted sensors during locomotion. Rather than claiming universal robustness under arbitrary dynamic loads, we focus on demonstrating that an online adaptation scheme can be executed in real time and can improve consistency in the recorded CoP trajectories within the scope of our walking experiments. In dynamic locomotion, the ground-projected CoM does not coincide with the CoP due to nonzero CoM acceleration, and stability only requires the CoP to remain within the contact convex envelope. Therefore, a self-calibration method that can operate during dynamic walking is needed. However, dynamic calibration also has practical limits: the centroidal terms used in the dynamic CoP model (e.g., CoM acceleration and the torso angular momentum rate) depend on IMU-based estimation and kinematic modeling and are sensitive to IMU noise, bias drift, and modeling errors. These uncertainties can propagate into the model-based CoP reference and affect calibration stability, especially during high-acceleration phases and contact transitions. In this work, we explicitly target normal walking regimes where these estimates remain sufficiently reliable, and we mitigate sensitivity by filtering the acceleration-related signals and restricting parameter update rates.

In addition, the optimization formulation is designed to reduce over-reliance on noisy model terms by (i) weighting residuals according to confidence, (ii) down-weighting or skipping frames with low vertical contact force (e.g., near lift-off), and (iii) enforcing smooth parameter evolution to avoid drift amplification. These design choices improve numerical stability while retaining the benefits of model-based self-calibration during dynamic walking.

The remainder of this article is organized as follows. Section 2 introduces the hardware design of the system and the design of the sensing system. Section 3 provides a detailed description of the CoP calibration method proposed in this paper. The experimental results are given in Section 4. Section 5 concludes this paper and suggests future work.

## 2. Design of Measurement System

### 2.1. Hardware Design of Measurement Sensing System

**Figure 1:**
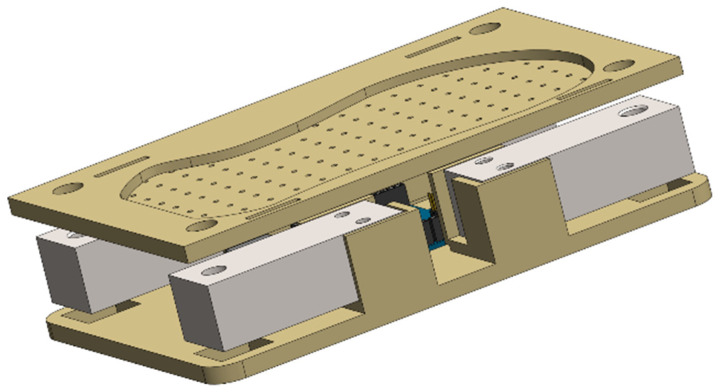
Model of the internal structure.

**Figure 2:**
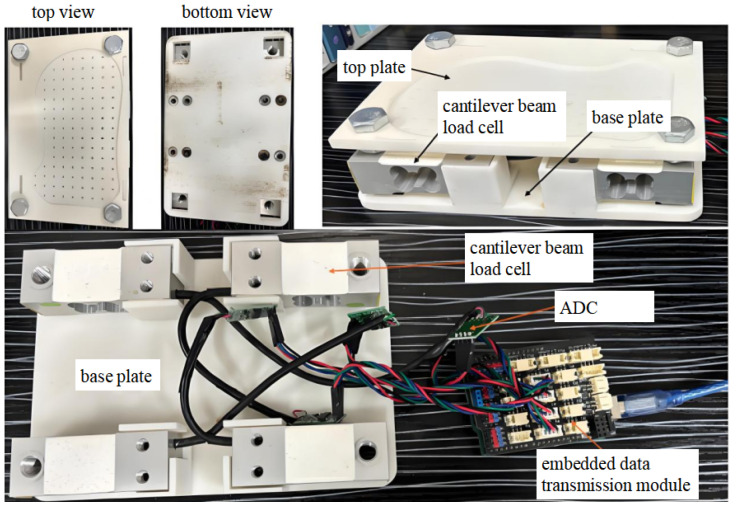
The internal hardware structure of the measuring device.

The fundamental principle of the measurement and sensing system is to utilize sensors to measure the CoP and vGRF during bipedal robot walking, thereby providing essential feedback for stability control. The system employs external cantilever beam-type load cells. A configuration of four load cells is deployed for each pressure measurement subsystem. The internal mechanical design of the measurement device is illustrated in Figure 1 and Figure 2.

The measurement and sensing system primarily consists of a top plate, cantilever beam load cells, and a base plate. The top plate connects to the sole of the NAO robot, securing one end of the cantilever beam sensors and allowing for precise adjustment via manual offline calibration. The cantilever beam load cells are used to measure vertical forces. They are connected by wires to an Analog-to-Digital Converter (ADC), which in turn is linked via wires to an embedded data transmission module. The base plate serves to anchor the opposite ends of the cantilever beam sensors and makes direct contact with the ground, ensuring overall system stability.

The four corners of the top plate are equipped with circular holes that align with the nut holes on the cantilever beam sensors, enabling secure fastening with nuts to ensure a rigid connection. The central portion of the top plate features an irregular polygonal shape that matches the original contour of the NAO’s foot sole, allowing the foot to be securely embedded into the plate. Additionally, the top plate incorporates small holes spaced 1 cm apart, enabling users to design and attach a weight cover. This facilitates manual offline calibration by providing concentrated stress points, thereby enhancing the measurement accuracy of the system.

The cantilever beam load cells are mounted onto the top plate via four small pedestals. Each sensor corner contains small holes for nut installation and tightening, ensuring stability during operation. Furthermore, the sensors are connected by wires to the ADC, which digitizes the sensor signals. These signals are then transmitted via wires to the embedded data transmission module for data acquisition and communication.

### 2.2. Design of Sensor Measurement System

**Figure 3:**
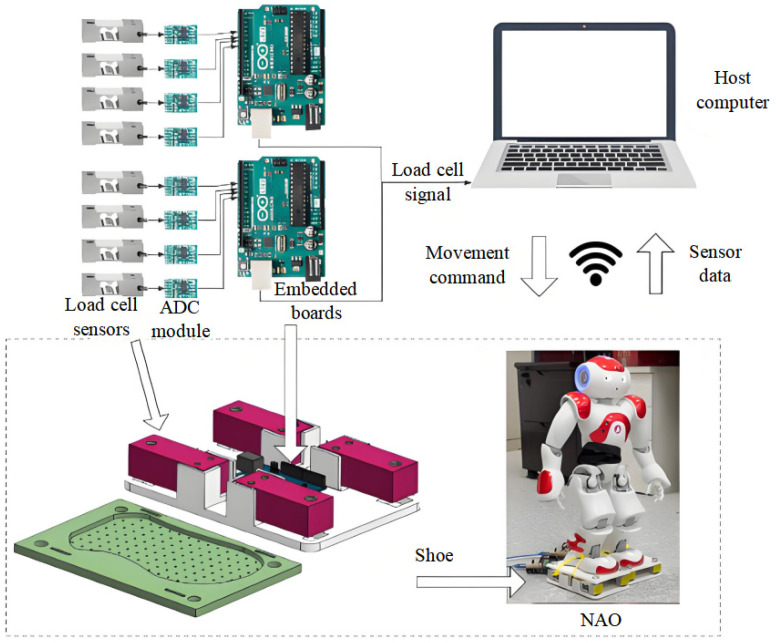
Sensor measurement system.

The basic idea of our framework is to fuse external foot-mounted sensing with the robot’s internal measurements to estimate CoP and the vGRF during bipedal walking, as shown in Figure 3. Each foot uses cantilever-beam load cells to convert vertical contact force into a small electrical signal (millivolt-level), which is amplified and digitized using an HX711-based ADC module. The digitized readings from four load cells are combined to reconstruct the resultant vertical contact force and compute the CoP on the host computer. Because the force signal is digitized and the offline calibration is performed using discrete load increments, the reconstructed vGRF can appear quantized (stepwise) in time plots; this reflects measurement resolution limits of the sensing chain rather than a discontinuity in the underlying physical force.

Although the load-cell–amplifier chain is commonly approximated as linear after offline calibration, it can exhibit non-ideal behaviors under dynamic loading, including hysteresis, creep, temperature-dependent drift, small cross-axis coupling, and contact-structure compliance effects. In this paper, we model the sensing chain using an effective affine mapping (scale and bias) for each channel, which captures the dominant drift and gain mismatch in practice. The proposed online self-calibration further adapts these effective parameters during walking, improving consistency under time-varying conditions; nevertheless, it does not claim to fully model all nonlinear or frequency-dependent sensor dynamics.

During operation, the host computer acquires the foot sensor readings via USB and simultaneously communicates with NAO through WLAN to receive internal states (e.g., IMU and kinematic information) and send motion commands. Using these external and internal measurements, the proposed online self-calibration module updates sensor scale and bias parameters in real time to obtain consistent CoP estimates during locomotion while also reconstructing a digitized vGRF estimate for CoP computation and model-consistency constraints.

The foot-sensor stream is acquired through USB at 80 Hz, by configuring the HX711 modules to the 80 SPS mode and time-stamping each sample on the host PC. The NAO internal sensor stream (IMU and kinematics used for centroidal terms) is queried at 100 Hz via NAOqi over WLAN and is also time-stamped on the host. To fuse both streams, we resample the NAO internal measurements to the 80 Hz foot-sensor timeline using linear interpolation. In our setup, the measured end-to-end communication delay for NAO-to-host telemetry is 20 ± 4 ms, while the USB acquisition delay is below 2 ms. A fixed-delay compensation of 20 ms is applied to align acceleration-related terms with the corresponding contact measurements, and all acceleration-related signals are low-pass filtered at 10 Hz to reduce sensitivity to timing jitter and IMU noise.

## 3. Calibration Method of CoP

### 3.1. Off-Line Manual Calibration Method

**Figure 4:**
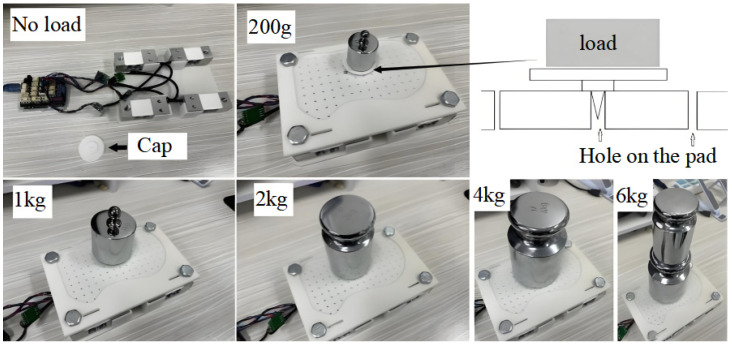
The offline manual calibration process.

To validate the consistency of the self-calibration algorithm, it is first necessary to obtain accurate CoP measurement signals through manual calibration. This section describes the offline manual calibration method used to verify the CoP self-calibration approach for dynamic motion CoP self-calibration method. The offline manual calibration process is shown in Figure 4.

For computational simplicity and ease of integration with the NAO foot structure, each foot is instrumented with four identical single-point sensors (Type: AT8541) arranged at the four corners of the foot plate, yielding eight channels in total. Prior to each experimental session, we perform an offline manual calibration using certified standard weights spanning 1 g–6 kg, covering the expected load range during NAO walking. For each channel, the no-load digital output *S_0_* is recorded, and additional outputs {*S_G_*} are collected under a set of known loads {*G*}. The channel-wise scale and bias coefficients are then identified by linear regression under an affine model *F = cS + d*. This weight-based procedure reduces inter-channel gain mismatch and offset drift before dynamic experiments and provides a traceable static reference for subsequent comparisons.

Step 1: Calibrate the sensors themselves. The no-load voltage *S*_0_ is recorded under zero load conditions (see “No Load” in Figure 4). Subsequently, the voltage *S_G_* is recorded under the application of known loads. Given the linear relationship between the output voltage of the FSR and the applied force, the linear coefficient can be derived as follows:

(1) $$\sigma \;=\;\left(S_{G}\;-\;S_{0}\right)/G$$

Therefore, the relationship between the applied force and the output voltage can be expressed as follows:

(2) $$F\;=\;\left(S_{G}\;-{\;S}_{0}\right)/\sigma \;=\;cS\;+\;d$$

where *c* and *d* are the scaling and biasing coefficients, respectively.

Step 2: Calibrate the measurement of CoP. A sample library is first constructed by applying weights of varying magnitudes to each calibration hole at a known, precise location. The calibration hole positions are defined as the true CoP${\left[Z_{\mathrm{real}}^{x},Z_{\mathrm{real}}^{y}\right]}^{T}$, and the system-computed CoP positions are regarded as the manually calibrated CoP${\;\left[\begin{array}{cc} z_{\mathrm{off}}^{x} & z_{\mathrm{off}}^{y} \end{array}\right]}^{T}$. The “off” subscript indicates offline calibration. As shown in Equation (3), the manually calibrated CoP${\;\left[\begin{array}{cc} z_{\mathrm{off}}^{x} & z_{\mathrm{off}}^{y} \end{array}\right]}^{T}$ is obtained by adding the CoP${\left[\begin{array}{cc} z_{0}^{x} & z_{0}^{y} \end{array}\right]}^{T}$ obtained from the original measurements with the corrected amount ${\left[\begin{array}{cc} \Delta x & \Delta y \end{array}\right]}^{T}$.

(3) $$\left[\begin{array}{c} z_{\mathrm{off}}^{x}\\ z_{\mathrm{off}}^{y} \end{array}\right]=\left[\begin{array}{c} z_{0}^{x}\\ z_{0}^{y} \end{array}\right]+\left[\begin{array}{c} \Delta x\\ \Delta y \end{array}\right]$$

where the amount of correction is defined as

(4) $$\left[\begin{array}{c} \Delta x\\ \Delta y \end{array}\right]=\left[\begin{array}{c} a_{1}z_{0}^{x2}+a_{2}z_{0}^{x}+a_{3}z_{0}^{y}+a_{4}+\sum_{i=1}^{4}m_{i}f_{i}\\ b_{1}z_{0}^{y2}+b_{2}z_{0}^{y}+b_{3}z_{0}^{x}+b_{4}+\sum_{i=1}^{4}m_{i}f_{i} \end{array}\right]$$

where $a_{j},b_{j}(j\;=\;1,2,3,4)$ and ${m_{i},n}_{i}(i\;=\;1,2,3,4)$ are the coefficients, $f_{i}$ is the contact force obtained from a single FSR measurement, and i is the sensor number. Here, the correction amount is composed of two parts. The first part is a second-order polynomial of the raw measurements with coefficients $a_{j},b_{j}(j\;=\;1,2,3,4)$, which serves to improve the average measured value at each calibration hole. The second part is a linear combination of first-order terms from individual force sensors, weighted by coefficients ${m_{i},n}_{i}(i\;=\;1,2,3,4)$, and is used to correct errors when different weights are applied to the same calibration hole. According to Equations (3) and (4), the error between the manually corrected CoP $z_{\mathrm{off}}$ and the true CoP${\;z}_{\mathrm{real}\;}≜\;{\left[\begin{array}{cc} z_{\mathrm{real}}^{x} & z_{\mathrm{real}}^{y} \end{array}\right]}^{T}$ is minimized using nonlinear least squares.

(5) $$\underset{\zeta }{\mathrm{argmin}}J=\sum_{k=1}^{N}{\left\|z_{\mathrm{real}}\left[k\right]-z_{\mathrm{off}}\left[k\right]\right\|}^{2}$$

where *k* is the sample index number, and $\zeta \;≜\;\left[a_{1},\;\cdots ,\;a_{4},\;b_{1},\;\cdots ,{\;b}_{4},{\;m}_{1},\;\cdots ,\;m_{4},{\;n}_{1},\;\cdots ,{\;n}_{4}\right]$ is the optimization variable. The optimal calibration coefficients can be obtained by solving Equation (5).

The polynomial correction model improves fitting accuracy on the offline calibration set, but it also increases model capacity and may overfit if the calibration samples do not sufficiently cover diverse loading distributions. To mitigate this risk, we construct the calibration library using multiple weight magnitudes and multiple hole locations, and we evaluate the fitted coefficients on held-out calibration placements. In addition, the subsequent online self-calibration stage adapts effective scale/bias parameters during walking, which helps compensate for residual mismatch between offline-fit coefficients and dynamic contact conditions.

### 3.2. CoP Dynamic Motion Model

**Figure 5:**
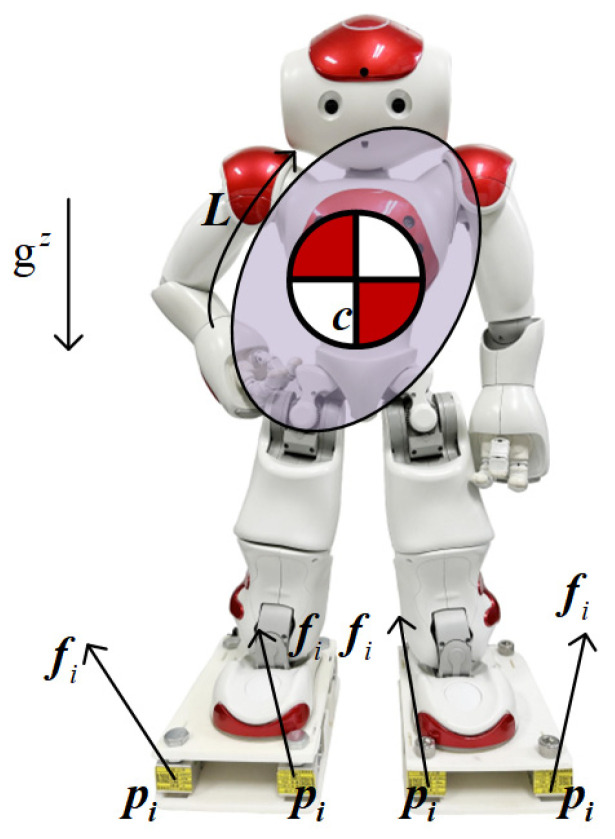
The CoP dynamic motion model for self-calibration.

This section presents the CoP dynamic motion model for self-calibration, as shown in Figure 5. For bipedal motion, the dynamical equations of the center-of-mass Newton-Euler form are

(6) $$m\left(\ddot{c}+g\right)=\sum_{i}f_{i}$$

(7) $$\mathrm{mc}\;\times \;\left(\ddot{c}+g\right)+\dot{L}=\sum_{i}\left(p_{i}-c\right)\;\times \;f_{i}$$

where m ∈ R denotes the total mass of the robot, $c\;=\;{\left[c^{x},c^{y},c^{z}\right]}^{T}$ denotes the position of the center of mass, and $g\;=\;{\left[0,0,g^{z}\right]}^{T}$ denotes the acceleration of gravity. $p_{i}\;={\;\left[p_{i}^{x},p_{i}^{y},p_{i}^{z}\right]}^{T}$ and $f_{i}\;=\;{\left[f_{i}^{x},f_{i}^{y},f_{i}^{z}\right]}^{T}$ denote the position of the *i*-th FSR and the ground contact force applied to the robot where it is located, respectively, $f_{i}^{z}$ is the output measured force of this FSR, and $L_{i\;}=\;{\left[L_{i}^{x},L_{i}^{y},L_{i}^{z}\right]}^{T}$ denotes the angular momentum of the robot torso with respect to the center of mass. *x*, *y*, *z* is used as a superscript to denote the corresponding axis of the physical quantities in a coordinate system, with the *x*-axis pointing in the forward direction of robot motion, the *z*-axis pointing in the direction of height opposite to the direction of gravitational acceleration, and the *y*-axis pointing in the direction specified by a right-handed system rule.

The center of pressure for bipedal locomotion is defined as

(8) $$z≜{\left[\begin{array}{cc} \frac{\sum_{i}f_{i}^{z}p_{i}^{x}}{\sum_{i}f_{i}^{z}} & \frac{\sum_{i}f_{i}^{z}p_{i}^{y}}{\sum_{i}f_{i}^{z}} \end{array}\right]}^{T}$$

The CoP computation is ill-conditioned when the total normal force is near zero (e.g., during foot swing or near lift-off/toe-off) because CoP is physically undefined without contact and numerically unstable due to division by a small Fz. Therefore, we compute CoP only for contact-valid samples that satisfy Fz > ε, with ε = 5 N (approximately 10% of NAO-V5 body weight, ≈53 N). For samples with Fz ≤ ε, the CoP is marked as invalid (not evaluated), and these samples are excluded from the CoP residual term in the online optimization so that division-amplified noise does not corrupt parameter updates. Importantly, this treatment does not mask instability: stability-related analysis (e.g., whether CoP remains within the support region) is evaluated only over contact-valid samples where CoP is physically meaningful. Swing-phase segments are explicitly treated as non-contact intervals rather than being interpreted as stable CoP measurements.

Defining the combined contact force on the soles of the robot’s feet in the height direction as $f_{N}\;≜\sum f_{i}^{z}$; by associating Equations (6)–(8), the dynamic motion model of bipedal motion CoP can be obtained:

(9) $$z^{x}=\;c^{x\;}-\;\frac{mc^{z}}{f_{N}}{\ddot{c}}^{x}\;-\;\frac{{\dot{L}}^{y}}{f_{N}}$$

(10) $$z^{y}={\;c}^{y}-\frac{mc^{z}}{f_{N}}{\ddot{c}}^{y}\;-\;\frac{{\dot{L}}^{x}}{f_{N}}\;$$

(11) $$f_{N}=m{\ddot{c}}^{z}+\mathrm{mg}$$

According to the CoP dynamic motion model in Equations (9)–(11), the model-based reference CoP and resultant vertical contact force can be computed in real time using the robot IMU and joint-encoder signals, enabling online self-calibration of the foot-sensor CoP measurements. This derivation assumes rigid foot–ground contact and a reliable mapping from sensor readings to vertical force distribution. In practice, foot compliance, uneven terrain contact, and partial contact during heel-strike/toe-off can violate these assumptions and introduce transient bias. To improve robustness, we incorporate contact-quality checks and confidence weighting in the optimization, and we restrict parameter update rates to prevent brief contact irregularities from causing large calibration changes.

### 3.3. Self-Calibration of CoP Measurements

This section provides a detailed explanation of the CoP self-calibration method. First, it outlines the parameter initialization method under static conditions, followed by dynamic self-calibration of the initialized parameters.

#### 3.3.1. Static Parameter Initialization

The CoP measurement self-calibration algorithm calibrates the c and d coefficients in Equation (2). First, we obtain reasonable initial values of parameters in the static case. To obtain reliable initial values of the scale and bias parameters in Equation (2), we perform an initialization phase under a quantitatively defined “quiet standing” condition. Specifically, the robot is commanded to a static posture (both feet on level ground) and remains stationary for Tinit seconds. To avoid transient effects after the posture command, the first Twarm seconds are discarded. From the remaining samples, we form a valid initialization set Kinit by enforcing both contact and motion-stability criteria:

(1) Contact validity: The estimated vertical contact force satisfies Fz > ε on each sample, ensuring that CoP-related quantities are well-conditioned.

(2) Motion stability: The magnitude of linear acceleration and angular velocity measured by the IMU are below thresholds, i.e., ||*a*|| < *a*th and ||ω|| < ωth, which rejects samples dominated by sway or brief disturbances.

We then solve the least squares initialization using all samples *k* ∈ Kinit (rather than a single instant), which reduces sensitivity to sensor noise and minor modeling mismatch. If |Kinit| is smaller than a minimum required number of samples, the standing interval is extended until sufficient valid samples are collected.

The relationship between the static-model-modeled quantities and the sensor-measured quantities is obtained by substituting the CoP definition in Equations (2) and (8):

(12) $$\left[\begin{array}{c} \mathrm{mg}\\ c^{x}\\ c^{y} \end{array}\right]\approx \left[\begin{array}{c} \sum_{i=1}^{8}\left(c_{0}S_{i}\;+{\;d}_{0}\right)\\ \sum_{i=1}^{8}\left(c_{0}S_{i}\;+{\;d}_{0}\right)p_{i}^{x}/\sum_{i=1}^{8}\left(c_{0}S_{i}\;+{\;d}_{0}\right)\\ \sum_{i=1}^{8}\left(c_{0}S_{i}\;+\;d_{0}\right)p_{i}^{y}/\sum_{i=1}^{8}\left(c_{0}S_{i}\;+{\;d}_{0}\right) \end{array}\right]$$

where $c_{0}$ and $d_{0}$ are the initial values of the scaling and biasing coefficients, respectively.

When more than one sample exists, Equation (12) can be expanded as follows:

(13) Y = AX

where, $Y=\left[\begin{array}{c} \vdots \\ c_{k}^{x}\mathrm{mg}\\ \begin{array}{c} c_{k}^{y}\mathrm{mg}\\ \vdots \end{array} \end{array}\right]$,$A=\left[\begin{array}{cc} \begin{array}{c} \vdots \\ \sum_{i=1}^{8}S_{k,i}p_{i}^{x}\\ \begin{array}{c} \sum_{i=1}^{8}S_{k,i}p_{i}^{y}\\ \vdots \end{array} \end{array} & \begin{array}{c} \vdots \\ \sum_{i=1}^{8}p_{i}^{x}\\ \begin{array}{c} \sum_{i=1}^{8}p_{i}^{y}\\ \vdots \end{array} \end{array} \end{array}\right]$,$X=\left[\begin{array}{c} c_{0}\\ d_{0} \end{array}\right]$.

Equation (13) is a typical linear equation-solving problem, which can be solved quickly and optimally using the least squares method.

#### 3.3.2. Real-Time Optimization of Dynamic Parameters

Following the acquisition of static initialization parameters, self-calibration is carried out in dynamic locomotion by minimizing the error between the experimental measurements (the vGRF and the CoP signal from the foot sensor system) and the theoretical outputs from the dynamic CoP motion model.

This is formulated as the following nonlinear optimization problem:

(14) $$\underset{\zeta }{\mathrm{argmin}}J\;=\sum_{k=1}^{N}\left({\left|f_{N}\left[k\right]\;-{\;f}_{N,\mathrm{model}}\right|}_{W_{f}}^{2}\;+{\;\left\|z\left[k\right]\;-{\;z}_{\mathrm{model}}\left[k\right]\right\|}_{W_{z}}^{2}\right)+\;{\left\|\zeta \;-{\;\zeta }_{0}\right\|}_{W_{\zeta }}^{2}$$

where *k* is the sample number, $f_{N,\mathrm{model}}$ and $z_{\mathrm{model}}$ are the combined ground contact force in the height direction and CoP position, respectively, computed according to the CoP dynamic motion model. $\zeta ≜\left[\left(c_{1},d_{1})\cdots (c_{8},d_{8}\right)\right]$ is the optimization variable, $\zeta_{0}$ is the initial guess value of the optimization variable, and $W_{f},W_{z},W_{\zeta }$ are the optimization weight matrices.

The weighting matrices balance trust between the foot-sensor measurements and the model-based references. To improve reproducibility and avoid arbitrary tuning, we use two steps.

First, we normalize residuals to remove unit dependence: the vertical-force residual is normalized by body weight, and the CoP residual is normalized by foot length *Lf.* This yields dimensionless residuals.

Second, we choose diagonal weights according to a confidence (inverse-variance) principle. Let δF denote the standard deviation of the normalized vertical-force measurement noise, estimated from the quiet-standing data (after offline calibration), and let δP denote the standard deviation of the normalized model-based CoP uncertainty induced by IMU/kinematics errors (estimated from the standing data and/or repeated trials). We then set

$$\omega_{F}\propto \frac{1}{{\delta F}^{2}}\;,\;\omega_{P}\propto \frac{1}{{\delta P}^{2}}$$

and use *ωS* as a regularization weight that enforces smooth evolution of the parameters between consecutive updates.

We further include a sensitivity study in which the main weights (*ωF*, *ωP*, *ωS*) are independently scaled within a broad range (e.g., ×0.2 to ×5), and we report the resulting variation in CoP/vGRF RMSE. The results show that performance varies smoothly with weights and remains stable over a wide interval, indicating that the method is not dependent on a single hand-tuned setting.

All residuals are normalized before weighting to improve reproducibility: the vertical-force residual is normalized by body weight, and the CoP residual is normalized by the foot length *Lf*. In our experiments, we use diagonal weights with fixed values.

$$\omega_{F}=1.0,\omega_{cop}=8.0,\omega_{\Delta \theta }=0.3$$

where $\omega_{F}$ weights the normalized vertical-force consistency term, $\omega_{\mathrm{cop}}$ weights the normalized CoP consistency term, and $\omega_{\Delta \theta }$ enforces smooth parameter evolution between consecutive updates. The nonlinear problem is solved online over a sliding window of 0.5 s. We update the solution at 20 Hz (every four samples) using a Gauss–Newton solver initialized from the previous solution, with five iterations per update. On the host PC, the average computation time per update is 2.5 ms, which supports real-time execution with ample margin.

To reduce sensitivity to unit scaling (Newton vs. meter), we normalize each residual by a nominal scale (e.g., body weight for vGRF and foot length for CoP) before weighting. We also bound parameter updates per iteration to improve numerical stability in real-time execution and to prevent occasional contact irregularities (e.g., foot slippage) from causing abrupt coefficient changes.

## 4. Experimental Validation

### 4.1. Experimental Environment and Parameter Settings

**Figure 6:**
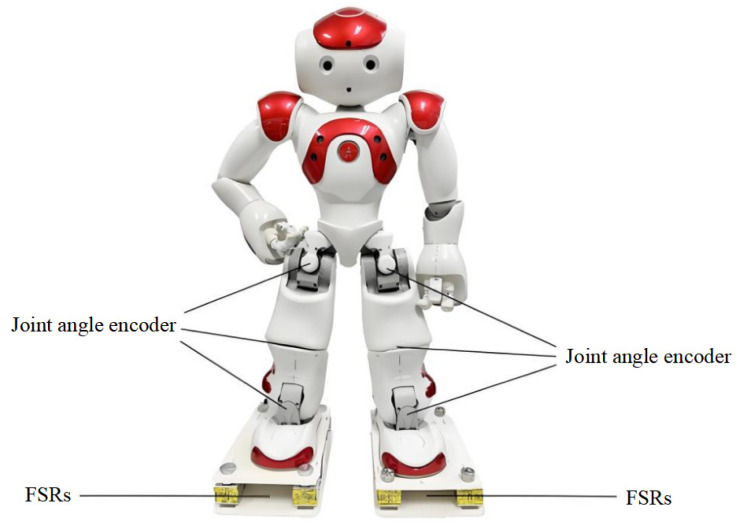
Sensors used in the experiment and their distribution.

This section conducts experiments using the Nao-V5 bipedal robot as a verification platform, where the robot’s motion is implemented through a built-in model-based predictive control gait algorithm. The relevant software and hardware configurations are detailed in Table 1, while the sensors and their distribution during the experiments are illustrated in Figure 6. The system undergoes precise calibration using the offline manual calibration method described in Section 3.1, with the obtained results serving as the reference values for the online self-calibration algorithm. Subsequently, online parameter optimization and calibration are performed.

Each load cell is read using an HX711 module with a 24-bit sigma–delta ADC and a gain of 128 (Channel A). The HX711 output data rate is configured to 80 SPS, and the host records synchronized samples at 80 Hz for each foot. The reconstructed vGRF therefore exhibits an effective discrete resolution; in our measurements the smallest observable increment in vGRF is approximately 0.5 N, which explains the stepwise appearance in some plots. To reduce high-frequency noise while retaining gait dynamics, we apply a zero-phase low-pass filter with a cutoff frequency of 10 Hz to the force-related signals. After time-stamping and stream alignment, the residual synchronization error is within ±5 ms, which is sufficient for the real-time use of acceleration-related terms in the proposed calibration objective.

### 4.2. Manual Offline Calibration

This section validates the manual offline calibration method presented in Section 3.1. The sensors are first calibrated individually. The outputs of the embedded data transmission module for the eight FSRs under no-load and various known load conditions are listed in Table 2. The scaling factors and offsets for all eight sensors, obtained by performing linear regression on the data in these tables, are presented in the last two columns of Table 2.

**Figure 7:**
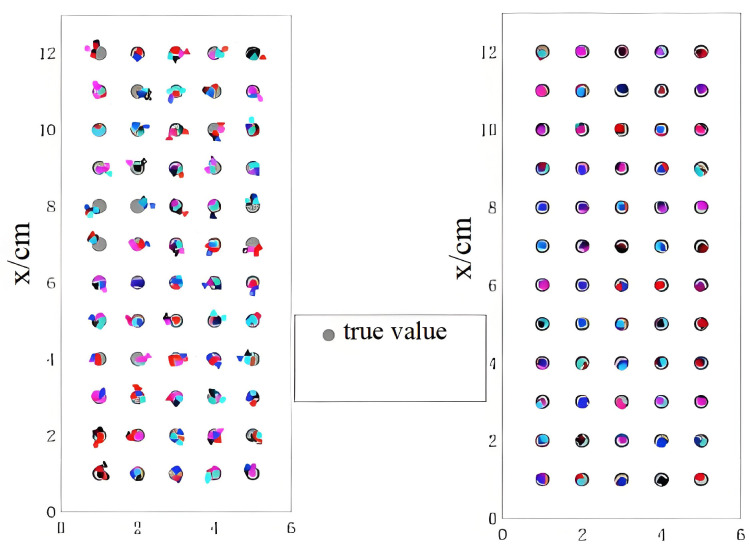
Manual offline calibration results of CoP.

**Figure 8:**
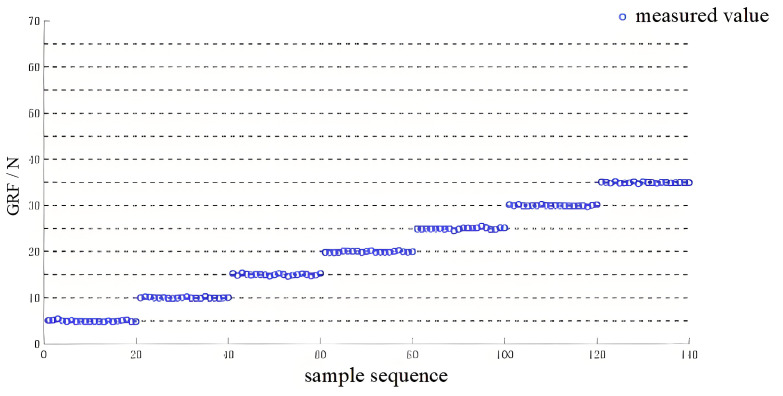
Manual offline calibration results of vGRF (digitized reconstruction; stepwise behavior may appear due to ADC quantization and discrete calibration loads).

The results of the manual offline calibration for the CoP and vGRF are shown in Figure 7 and Figure 8, respectively. The RMSE obtained using the initial coefficients and the corrected coefficients is reported in Table 3. The corrected CoP values are much closer to the reference positions (gray circles) than the initial measurements, and the reconstructed vGRF signals show improved agreement with the offline-calibrated reference curves (black dotted lines). Therefore, in the remainder of this paper, we use the manual offline calibration results as a reference baseline for evaluating the proposed online self-calibration method during dynamic walking. We note that this baseline is not an independent metrology reference (e.g., a force plate), since it is obtained using the same sensing hardware; thus, the evaluation primarily reflects consistency and improvement relative to the offline-calibrated baseline.

The offline calibration results are used as a reference baseline rather than an independent ground truth. To quantify the reliability of this baseline, we repeat the offline placement measurements 20 times at representative calibration holes and loads and report mean ± standard deviation for the corresponding CoP coordinates. These uncertainty bounds are included in the revised manuscript to contextualize millimeter-level improvements observed during online calibration.

### 4.3. Online Parameter Optimization

**Figure 9:**
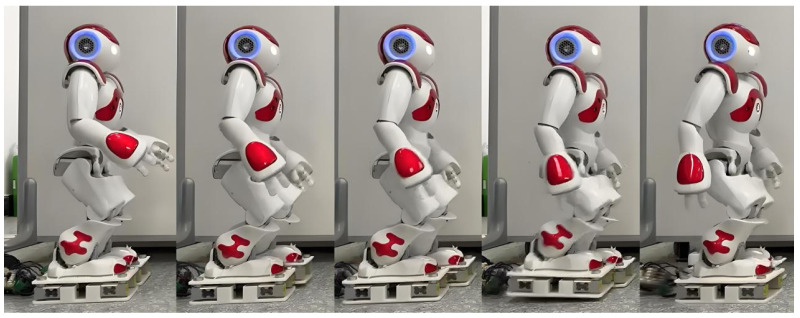
Video screenshots during the motion.

This section presents the validation of the proposed CoP measurement system during dynamic locomotion, with a comparative evaluation against the existing Han’s method [31] that relies on a static motion assumption. The experiment was conducted under dynamic conditions, and the video screenshots during the motion are shown in Figure 9.

**Figure 10:**
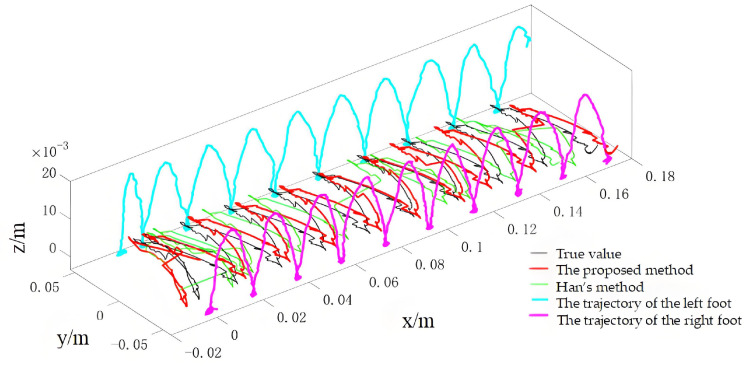
Online self-calibration results of CoP during dynamic walking at 0.02 m/s (compared against the offline-calibrated reference baseline).

**Figure 11:**
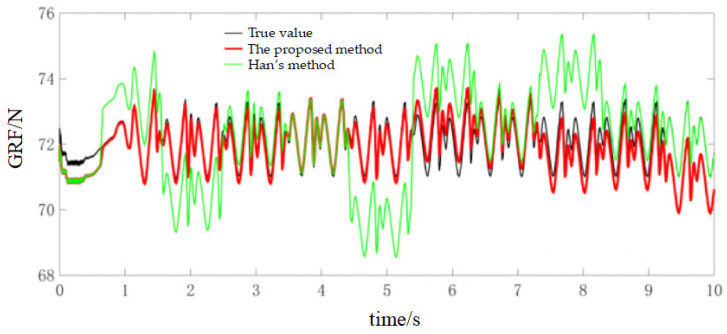
Online self-calibration results of vGRF at 0.02 m/s (digitized reconstruction; stepwise behavior may appear due to ADC quantization).

**Figure 12:**
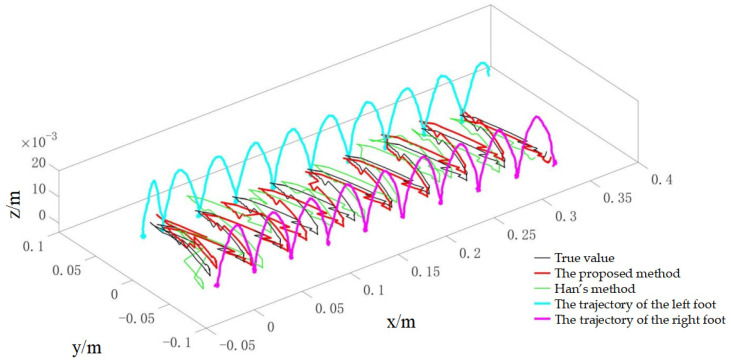
Online self-calibration results of CoP during dynamic walking at 0.04 m/s (compared against the offline-calibrated reference baseline).

**Figure 13:**
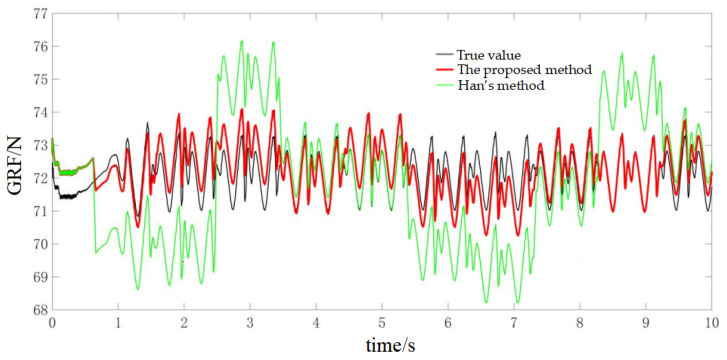
Online self-calibration results of vGRF at 0.04 m/s (digitized reconstruction; stepwise behavior may appear due to ADC quantization).

**Figure 14:**
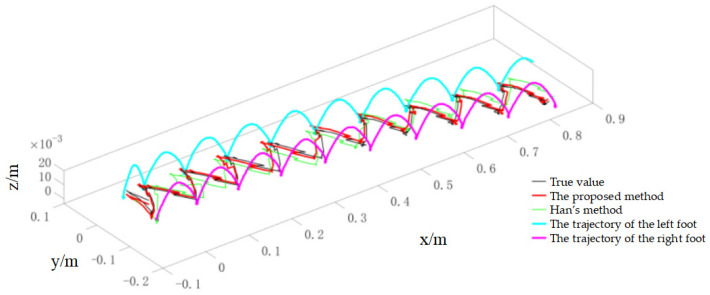
Online self-calibration results of CoP during dynamic walking at 0.1 m/s (compared against the offline-calibrated reference baseline).

**Figure 15:**
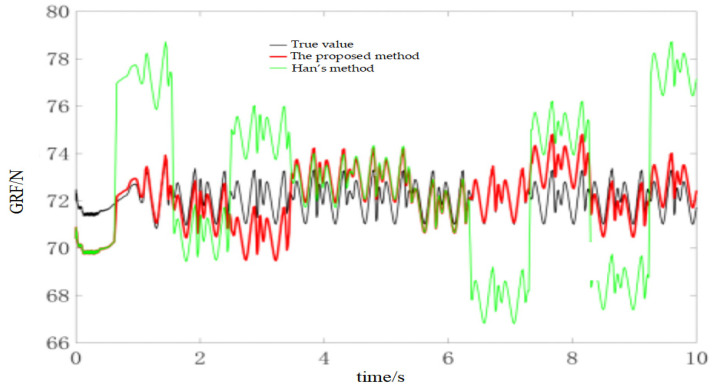
Online self-calibration results of vGRF at 0.1 m/s (digitized reconstruction; stepwise behavior may appear due to ADC quantization).

During the experiments, the robot walked on level ground at three speeds (0.02 m/s, 0.04 m/s and 0.1 m/s) to evaluate the method under different dynamic conditions. For the 0.02 m/s condition, the online calibration results for CoP and vGRF are presented in Figure 10 and Figure 11, respectively, with the corresponding RMSE values summarized in Table 4. For the 0.04 m/s condition, the respective results are shown in Figure 12 and Figure 13, and the RMSE values are provided in Table 5. For the 0.1 m/s condition, the respective results are shown in Figure 14 and Figure 15, and the RMSE values are provided in Table 6.

For quantitative comparison, we report errors with respect to the offline-calibrated reference baseline (Section 4.2) and compare against Han’s method [31], which relies on a static motion assumption. The results across all speeds indicate that the proposed method yields CoP trajectories that are more consistent with the offline reference baseline than the static-assumption method, and the reconstructed vGRF shows reduced deviation relative to the same baseline (noting that the vGRF signal may appear stepwise due to digitization/quantization).

These preliminary results demonstrate the potential of the proposed online method to improve estimation consistency during dynamic walking. However, the current evaluation remains limited in terms of long-term and systematic characterization. Future work should include: (i) quantitative analysis of parameter convergence behavior (e.g., time-to-convergence, steady-state variance, and scale/bias drift), (ii) assessment of long-term stability over extended walking durations, and (iii) repeatability evaluation across multiple trials and gait cycles. Such analyses would require longer recordings, repeated experiments, and controlled variations in walking conditions to distinguish estimator behavior from trial-to-trial variability.

While the present study validates the method on a single NAO-V5 platform under level-ground walking at multiple speeds, broader scenarios—such as gait transitions (start/stop/turn) and external disturbances—should be investigated to further assess generality. Although within-run repeatability is quantified via gait cycle statistics in this work, future experiments should incorporate independent multi-session trials to better characterize between-trial variability and strengthen statistical conclusiveness.

## 5. Discussion

### 5.1. Failure Modes and Operational Boundaries

The proposed online self-calibration relies on a dynamic reference model that implicitly assumes: (i) rigid contact between the foot and the ground, with negligible foot/ground compliance and no slip, and (ii) sufficiently reliable IMU-derived acceleration after filtering and time alignment. These assumptions are reasonable for moderate-speed walking on rigid, flat terrain, but can be violated under more dynamic conditions. Below we summarize the main failure modes and practical operating boundaries.

(1) Non-rigid contact and compliance.

If the foot sole or the ground exhibits significant compliance, the contact pressure distribution and CoP shift can deviate from the rigid-contact model. This may produce systematic residuals that the optimizer can incorrectly attribute to sensor scale/bias, causing parameter drift. Symptoms include persistent CoP bias during stance and load-dependent hysteresis.

(2) Slip, partial contact, and rolling contact.

Slip or partial contact violates the assumed static friction constraint and changes the mapping from sensor forces to CoP. Under slip, the measured CoP may move rapidly without a corresponding kinematic explanation, potentially destabilizing the update. Rolling contact (heel-to-toe) can also amplify transient errors near contact transitions.

(3) Impact transients and high-frequency dynamics.

Heel-strike impacts introduce short-lived acceleration spikes and high-frequency vibrations. IMU acceleration estimates in this regime can be corrupted by sensor saturation, aliasing, or insufficient bandwidth. If such transients are directly used in the residual, the optimizer may overreact, leading to abrupt parameter changes.

(4) IMU bias, misalignment, and synchronization errors.

IMU bias and orientation errors cause acceleration estimation errors, while time misalignment between IMU and foot sensors can create apparent model mismatch. These errors can accumulate and lead to consistent residuals that are not caused by sensor gain/offset, again risking biased calibration updates.

(5) Low normal force and CoP ill-conditioning.

Near heel-strike and toe-off, the vertical force becomes small, making CoP estimation ill-conditioned and highly sensitive to noise. In this regime, CoP residuals can be dominated by noise rather than physical error.

### 5.2. Safeguards and Operational Boundaries

To mitigate the above issues, we adopt several practical safeguards:

CoP-related residuals are evaluated only when the estimated normal force exceeds a threshold; otherwise, CoP is held or down-weighted;

Acceleration-related terms are low-pass filtered and down-weighted when IMU confidence is low;

Parameter updates are rate-limited or regularized to prevent overreaction to transient outliers;

Outlier rejection is applied around contact transitions.

These measures improve numerical stability within the tested conditions; however, they do not provide a guarantee under severe impacts, strong compliance, or large disturbances.

The current implementation assumes rigid contact and sufficiently accurate IMU-based acceleration after filtering and synchronization. Under highly dynamic locomotion (strong impacts), compliant terrain, slip, or significant IMU bias/saturation, these assumptions may be violated, and the optimizer may attribute model mismatch to sensor parameters. Therefore, while the method shows improved within-trial consistency under the tested conditions, its robustness boundaries under severe dynamics remain to be characterized. Future work will incorporate compliant-contact modeling and improved inertial estimation (e.g., bias tracking and sensor fusion) and evaluate performance under controlled disturbances and varied terrains.

## 6. Conclusions

This study designed a foot-mounted CoP measurement system for dynamic bipedal walking and developed an online self-calibration method that updates sensor scale and bias parameters without manual intervention during locomotion. Experiments on a NAO-V5 robot demonstrated that, compared with a baseline method relying on a static motion assumption, the proposed approach provides CoP estimates more consistent with an offline-calibrated reference baseline under dynamic walking conditions. The same framework also reconstructs a digitized estimate of the vertical ground reaction force (vGRF) from load-cell measurements. Due to sensing/ADC resolution and the discrete nature of offline calibration loads, the vGRF signal may exhibit quantization and should be interpreted as a reconstructed digital signal rather than laboratory-grade continuous force metrology.

In this work, manually offline-calibrated results served as the reference baseline for evaluating the online self-calibration during dynamic walking. Since both the baseline and the online method rely on the same sensing hardware, the reported RMSE values primarily reflect relative improvement and self-consistency with respect to the offline reference, not absolute metrological accuracy against an independent gold-standard system such as a force plate or a high-precision force/torque sensor. Therefore, the improvements reported here are interpreted as enhanced consistency, robustness, and reduced drift/mismatch during dynamic locomotion, without claiming force plate-level absolute accuracy.

Future work will focus on deeper mechanical integration with the robot foot to reduce weight and improve dynamic performance, as well as broader validation across gait transitions (start/stop/turn) and disturbance scenarios. In addition, we plan to evaluate absolute force accuracy using an independent reference system (e.g., a force plate or a commercial force/torque sensor) to quantify metrology-level performance beyond consistency with the offline-calibrated baseline. Furthermore, to enable rigorous statistical validation, follow-up studies will involve multiple repeated trials per walking condition (including different speeds), reporting of means ± standard deviations (or confidence intervals), and statistical testing to assess the significance of performance improvements.

## Figures and Tables

**Table 1:** Related software and hardware environment configuration.

Name	Model
load cell	Autoda AT8541
ADC	Avia Semiconductor HX711
embedded controller	Arduino UNO
wireless router	Mercury D19G
host computer	NUC NUC14RVH

**Table 2:** The outputs of the embedded data transmission module.

Load Number	No Load	0.01 kg	0.05 kg	0.2 kg	Scaling Coefficients	Biasing Coefficients
left foot, front left	8,347,750	8,344,582	8,326,926	8,264,253	−2.38250654 × 10^−6^	19.8895457
left foot, front right	8,347,750	8,344,582	8,301,298	8,264,254	−2.20853048 × 10^−6^	18.4277626
left foot, rear left	8,322,914	8,317,325	8,301,301	8,264,263	−3.47692594 × 10^−6^	28.9285295
left foot, rear right	8,322,914	8,317,325	8,301,299	8,264,278	−3.47779548 × 10^−6^	28.9357592
right foot, front left	8,322,914	8,317,426	8,301,297	8,264,254	−3.47368720 × 10^−6^	28.9017197
right foot, front right	8,322,914	8,317,356	8,301,289	8,264,261	−3.47589563 × 10^−6^	28.9199912
right foot, rear left	8,322,914	8,317,320	8,301,299	8,264,262	−3.47698378 × 10^−6^	28.9290027
right foot, rear right	8,321,583	8,317,321	8,301,297	8,264,267	−3.52997190 × 10^−6^	29.3677097

**Table 3:** Manual offline calibration RMSE.

Value	Initial Value	After Manual Offline Calibration
CoP (m)	0.1839	0.0005
vGRF (N)	5.298	0.08

**Table 4:** Algorithm RMSE at 0.02 m/s.

	CoP RMSE (m)	vGRF RMSE (N)
The proposed method	0.0059	0.4339
Han’s method	0.0108	1.2949

**Table 5:** Algorithm RMSE at 0.04 m/s.

	CoP RMSE (m)	vGRF RMSE (N)
The proposed method	0.0108	0.5324
Han’s method	0.0166	1.8646

**Table 6:** Algorithm RMSE at 0.1 m/s.

	CoP RMSE (m)	vGRF RMSE (N)
The proposed method	0.0134	1.0387
Han’s method	0.0199	2.5220

## Data Availability

The original contributions presented in this study are included in the article. Further inquiries can be directed to the corresponding authors.
